# Investigating the Role of Diet and Exercise in Gut Microbe-Host Cometabolism

**DOI:** 10.1128/mSystems.00677-20

**Published:** 2020-12-01

**Authors:** N. Penney, W. Barton, J. M. Posma, A. Darzi, G. Frost, P. D. Cotter, E. Holmes, F. Shanahan, O. O’Sullivan, I. Garcia-Perez

**Affiliations:** a Division of Surgery, Department of Surgery and Cancer, Faculty of Medicine, Imperial College London, London, United Kingdom; b APC Microbiome Ireland, University College Cork, National University of Ireland, Cork, Ireland; c Teagasc Food Research Centre, Moorepark, Co. Cork, Ireland; d Department of Medicine, University College Cork, National University of Ireland, Cork, Ireland; e Section of Bioinformatics, Division of Systems Medicine, Department of Metabolism, Digestion and Reproduction, Faculty of Medicine, Imperial College London, London, United Kingdom; f Health Data Research UK, London, United Kingdom; g Section for Nutrition Research, Division of Digestive Diseases, Department of Metabolism, Digestion and Reproduction, Faculty of Medicine, Imperial College London, London, United Kingdom; Pacific Northwest National Laboratory

**Keywords:** diet, exercise, metabolism, microbiome

## Abstract

We investigated the individual and combined effects of diet and physical exercise on metabolism and the gut microbiome to establish how these lifestyle factors influence host-microbiome cometabolism. Urinary and fecal samples were collected from athletes and less active controls. Individuals were further classified according to an objective dietary assessment score of adherence to healthy dietary habits according to WHO guidelines, calculated from their proton nuclear magnetic resonance (^1^H-NMR) urinary profiles. Subsequent models were generated comparing extremes of dietary habits, exercise, and the combined effect of both. Differences in metabolic phenotypes and gut microbiome profiles between the two groups were assessed. Each of the models pertaining to diet healthiness, physical exercise, or a combination of both displayed a metabolic and functional microbial signature, with a significant proportion of the metabolites identified as discriminating between the various pairwise comparisons resulting from gut microbe-host cometabolism. Microbial diversity was associated with a combination of high adherence to healthy dietary habits and exercise and was correlated with a distinct array of microbially derived metabolites, including markers of proteolytic activity. Improved control of dietary confounders, through the use of an objective dietary assessment score, has uncovered further insights into the complex, multifactorial relationship between diet, exercise, the gut microbiome, and metabolism. Furthermore, the observation of higher proteolytic activity associated with higher microbial diversity indicates that increased microbial diversity may confer deleterious as well as beneficial effects on the host.

**IMPORTANCE** Improved control of dietary confounders, through the use of an objective dietary assessment score, has uncovered further insights into the complex, multifactorial relationship between diet, exercise, the gut microbiome, and metabolism. Each of the models pertaining to diet healthiness, physical exercise, or a combination of both, displayed a distinct metabolic and functional microbial signature. A significant proportion of the metabolites identified as discriminating between the various pairwise comparisons result from gut microbe-host cometabolism, and the identified interactions have expanded current knowledge in this area. Furthermore, although increased microbial diversity has previously been linked with health, our observation of higher microbial diversity being associated with increased proteolytic activity indicates that it may confer deleterious as well as beneficial effects on the host.

## Abstract (precis)

Improved control of dietary confounders, through the use of an objective dietary assessment score, has uncovered further insights into the complex, multifactorial relationship between diet, exercise, the gut microbiome, and metabolism. Each of the models pertaining to diet healthiness, physical exercise, or a combination of both, displayed a distinct metabolic and functional microbial signature.

## INTRODUCTION

Recent studies have shown that increased physical activity and aerobic fitness may mediate some health benefits through modulation of the gut microbiome (1–4). Human studies have shown differences in the gut microbiome, including increased diversity, in both habitual exercisers (5, 6) and professional athletes (7–10) compared to more sedentary controls. Longitudinal studies have also found exercise-dependent compositional and functional changes in the gut microbiome that result in altered levels of a number of microbially derived bioactive metabolites such as short-chain fatty acids (SCFA) as well as improved glucose homeostasis (7, 11, 12).

However, understanding the relationship between the gut microbiome, diet, and exercise remains elusive in part because of the dietary adaptations that accompany habitual physical activity. Difficulties understanding this relationship are compounded by inaccuracies in assessing dietary habits, with 30 to 88% misreporting using traditional self-reported assessment tools (13–15). In an earlier report, taxonomic and functional diversity were positively correlated with both dietary protein intake and physical exercise in a population of athletes (7, 8). We hypothesized that more accurate dietary assessment would help separate the contributions of diet and exercise to the modulation of the gut microbiota and enhance our understanding of the contribution of diet and exercise to the modulation of microbe-host cometabolism. To achieve this, we applied metabolic profiling combined with a mathematical modeling strategy to provide objective evidence of adherence to World Health Organization (WHO) healthy eating guidelines (increased fruits, vegetables, whole grains, and dietary fiber and decreased fats, sugars, and salt) (16, 17). Our approach characterizes volunteers more accurately according to physical activity and dietary status. By minimizing dietary variation in volunteer subsets the study has uncovered insights into the distinct influences of physical activity and dietary status on host metabolism, the gut microbiome, and subsequent gut microbiome-host cometabolism. In addition, we have explored the functional and metabolic implications of higher microbial diversity, previously reported in athletes (7, 8), on the host.

## RESULTS

**FIG 1:**
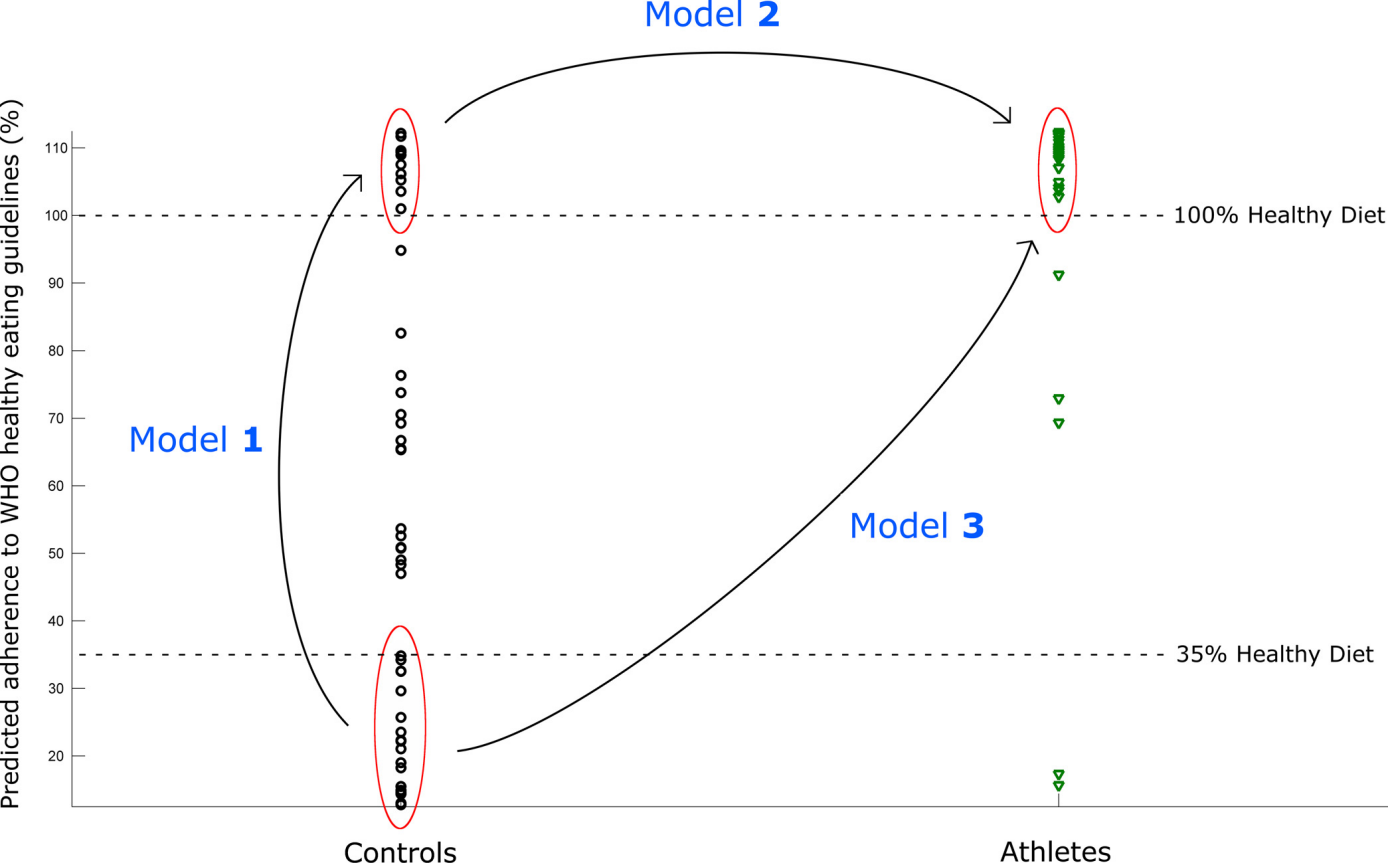
Clustering of individuals according to exercise status and adherence to healthy eating guidelines. Predicted adherence to WHO healthy eating guidelines calculated from ^1^H-NMR urinary profile of each individual using a validated metabolic profiling tool (see Materials and Methods for dietary assessment methodology). Individuals were subsequently clustered to form comparative models. Model 1 (healthy diet effect): comparing controls with a <35% adherence versus controls with a ≥100% adherence to WHO healthy eating guidelines. Model 2 (effect of exercise): controls with a ≥100% adherence versus professional athletes with a ≥100% adherence to WHO healthy eating guidelines. Model 3 (combined diet and exercise effect): controls with a <35% adherence versus athletes with a ≥100% adherence to WHO healthy eating guidelines.

To assess the separate contributions of diet and exercise in modulating the gut microbiota, we investigated urine and fecal samples from male athletes (*n* = 40) in the national Irish Rugby Football team that were collected while attending an intensive training camp and healthy controls (*n* = 46) matched for age and gender, as previously described (8). We used a previously validated, metabolic profiling dietary assessment tool (17) to objectively assess and score participants’ dietary habits (Fig. 1). Higher calculated scores indicate more complete adherence to WHO dietary guidelines of increased fruits, vegetables, whole grains, and dietary fiber and decreased fat, sugar, and salt consumption. Briefly, the dietary profiling model was built using proton nuclear magnetic resonance (^1^H-NMR) global urinary metabolic profiles derived from healthy participants that attended an inpatient randomized controlled crossover trial and were assigned to diets with differing levels of adherence to WHO healthy eating guidelines (16, 17) (see Materials and Methods).

As expected, controls had diverse adherence to a healthy diet, while professional athletes were predominantly assigned scores that met or exceeded the healthy eating guidelines with healthy eating scores of ≥100%, reflecting the known association between habitual exercise and good dietary habits (18). The scores for healthy eating behavior for the less active control participants ranged from 12.8% to 112.2%, with a relatively even distribution between the two extremes, while those of the athletes ranged from 15.7% to 112.5%, with only 5 of the 40 professional athlete participants falling below the 100% boundary. Individuals were then stratified according to their score to construct comparative groups (Fig. 1). Participants with a scored adherence to WHO healthy eating guidelines of ≥100% were considered healthy eaters. A cluster of controls was observed with a healthy eating adherence score of <35%. There is evidence that those at the bottom third of a healthy eating scale have a 25% higher all-cause mortality than those in the top third, as well as a 40% higher mortality from cardiovascular disease (19). Therefore, 35% was chosen as the cutoff to define unhealthy eaters. We used these groupings to construct a variety of models to statistically measure the differences in host metabolism, the gut microbiome, and their subsequent cometabolism between various groups (Fig. 1). Model 1 (healthy diet effect) was constructed comparing controls with a <35% adherence (*n* = 19) versus controls with a ≥100% adherence to healthy eating (*n* = 11); model 2 (exercise effect) compared controls with a ≥100% adherence to healthy eating (*n* = 11) versus athletes with a ≥100% adherence to healthy eating (*n* = 35); model 3 (combined healthy diet and exercise effect) compared controls with a <35% adherence to healthy eating (*n* = 19) versus athletes with a ≥100% adherence to healthy eating (*n* = 35). Other lifestyle factors, including smoking levels and alcohol consumption, were also measured. The were no significant differences in these factors between groups.

### Diet and exercise status were associated with distinct urinary and fecal metabolomes.

**FIG 2:**
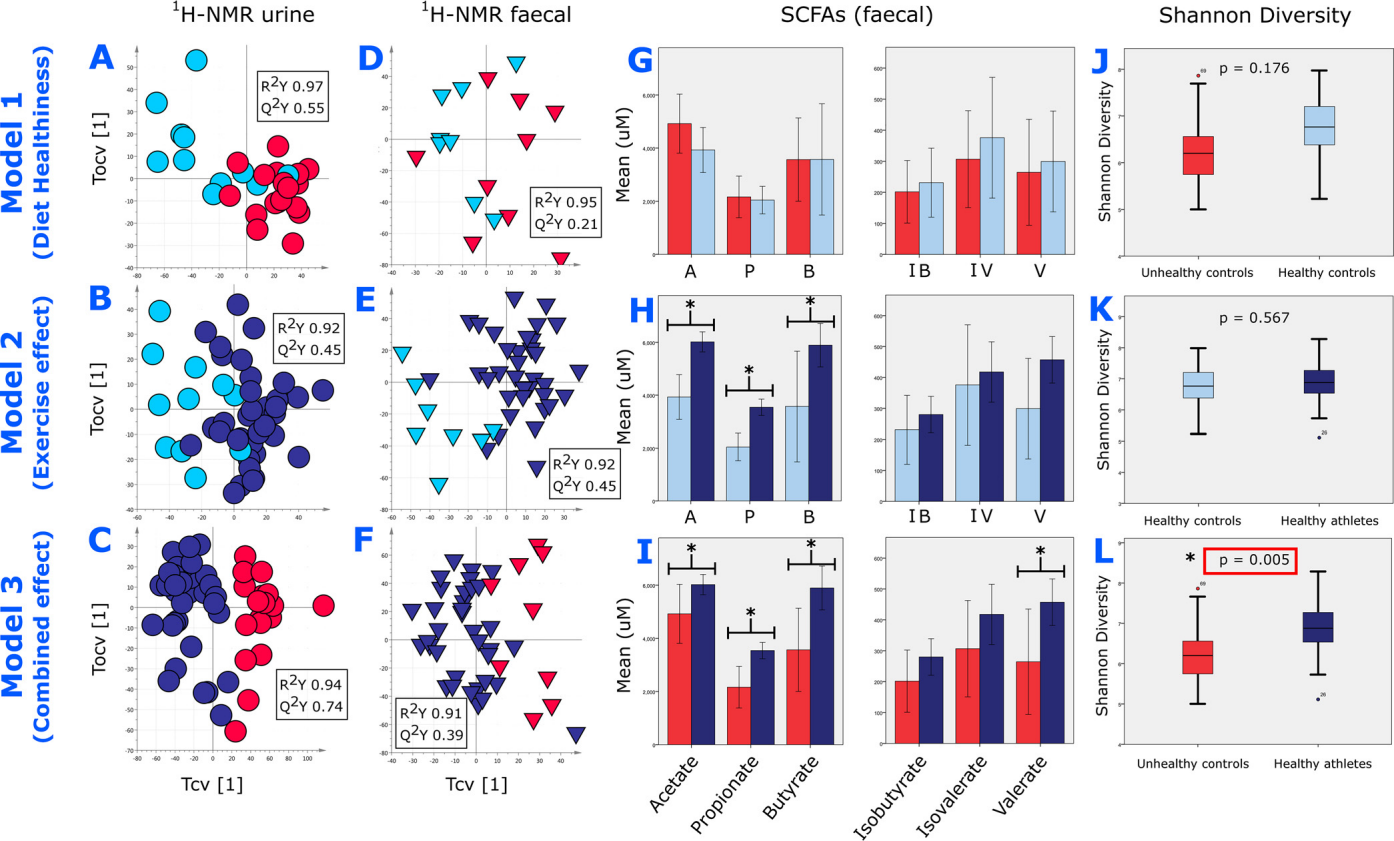
^1^H-NMR metabolic phenotyping, SCFA, and microbial diversity results. (A to F) Cross-validated OPLS-DA score plots, generated with one predictive (Tcv) and one orthogonal (Tocv) component, of ^1^H-NMR urinary profiles comparing (A) controls with a <35% adherence (red) to controls with a ≥100% adherence (light blue) to WHO healthy eating guidelines (model 1, healthy diet effect), (B) controls with a ≥100% adherence to healthy eating (light blue) to professional athletes with a ≥100% adherence to healthy eating (dark blue) (model 2, exercise effect), (C) controls with a <35% adherence to healthy eating (red) versus professional athletes with a ≥100% adherence to healthy eating (dark blue) (model 3, combined effect), and ^1^H-NMR fecal profiles comparing (D) model 1, healthy diet effect, (E) model 2, exercise effect, and (F) model 3, combined effect. (G to I) Bar charts of mean SCFA levels measured through quantitative GC-MS comparing (G) model 1, healthy diet effect, (H) model 2, exercise effect, and (I) model 3, combined effect. (J to L) Box plots showing mean Shannon diversity index levels of taxa described by 16S profiling comparing (J) model 1, healthy diet effect, (K) model 2, exercise effect, and (L) model 3, combined effect. The 95% confidence intervals shown. Significant results (pFDR,  <0.05) are marked with *.

Based on these comparative groups, six statistically robust Orthogonal Projections to Latent Structures Discriminant Analysis (OPLS-DA) models (Fig. 2A to F) were obtained to assess the effects of diet, exercise, and the two in combination on participants’ urinary and fecal metabolic profiles, measured by ^1^H-NMR. The urinary model characterizing the combined effect of diet healthiness and exercise was strongest (model 3; Fig. 2C, R^2^_Y_ = 0.94, Q^2^_Y_ = 0.74), but statistically robust urinary models were also obtained defining the impact of diet healthiness (model 1; Fig. 2A, R^2^_Y_ = 0.97, Q^2^_Y_ = 0.55) and exercise alone (model 2; Fig. 2B, R^2^_Y_ = 0.92, Q^2^_Y_ = 0.45). With regard to fecal profiles, the strongest model was that defining the effect of exercise (model 2; Fig. 2E, R^2^_Y_ = 0.92, Q^2^_Y_ = 0.45). The combination of diet healthiness and exercise produced a relatively robust model (model 3; Fig. 2F, R^2^_Y_ = 0.91, Q^2^_Y_ = 0.39), while investigation of diet alone showed the least impact (model 1; Fig. 2D, R^2^_Y_ = 0.95, Q^2^_Y_ = 0.21).

For each pairwise OPLS-DA model built with the global ^1^H-NMR metabolic profiles, a number of metabolites discriminating between the two classes were identified from the model coefficients. These are listed in Table S1 (urine) and Table S2 (feces). As expected, in model 1 (healthy diet effect), urinary metabolites with well-known dietary associations such as proline-betaine (found in citrus fruit, particularly oranges [20]) and hippurate (associated with the consumption of fruits and vegetables) were significantly higher in the group with healthier eating scores, whereas markers of oxidative stress (2-hydroxybutyrate) and anaerobic metabolism (lactate) were found in lower concentrations. Fecal metabolic profiles in subjects with healthier diet scores were associated with lower concentrations of 2-aminobutyrate and 2-hydroxybutyrate and higher levels of markers of cruciferous vegetable intake (*S*-methyl-l-cysteine-sulfoxide derivatives). In the exercise effect model (model 2), the athlete group was characterized by a number of urinary metabolites derived from the gut microbiome, such as phenylacetylglutamine (PAG) and 3-indoxyl sulfate (3IS), as well as the ketone body acetoacetate and markers of red meat intake and fatty-acid (FA) metabolism (carnitine, *O*-acetyl carnitine). In contrast, markers of oxidative stress (allantoin [21]) and tricarboxylic acid (TCA) cycle intermediates (succinate and citrate) were higher in the control group. Analysis of fecal metabolites revealed higher concentrations of the short-chain fatty acids (SCFAs) acetate, propionate, butyrate, and valerate and lower levels of the amino acids glycine, phenylalanine, and tyrosine in the athlete group compared to controls consuming a comparatively healthy diet. Model 3 (combined effect) consisted predominantly of metabolites derived from models 1 and 2. Healthy eating, exercise, and the combined effect of both (models 1 to 3) were all characterized by higher markers of choline metabolism, with higher urinary trimethylamine-*N*-oxide (TMAO). In the exercise effect model (model 2), this corresponded with higher fecal methylamine and trimethylamine and lower dimethylamine in athletes relative to controls.

10.1128/mSystems.00677-20.6 TABLE S1 Urinary metabolites discriminating between model classes. Tables show significantly higher or lower levels of urinary metabolites when comparing two different groups. Model 1 (adherence to WHO healthy eating guidelines): controls only comparing individuals with a <35% adherence versus those with a ≥100% adherence to WHO healthy eating guidelines. Model 2 (exercise): controls with a ≥100% adherence versus athletes with a ≥100% adherence to WHO healthy eating guidelines. Model 3 (combined diet and exercise effect): controls with a <35% adherence versus athletes with a ≥100% adherence to WHO healthy eating guidelines. ^a^Multiplicity key is as follows: s, singlet; d, doublet; t, triplet; q, quartet; dd, doublet of doublets; m, multiplet. ^1^H shifts marked by an asterisk (*) were not confirmed experimentally due to the absence of unambiguous resonance values and are taken from the Human Metabolome Database (HMDB). Download Table S1, DOCX file, 0.03 MB. Copyright © 2020 Penney et al. 2020 Penney et al. This content is distributed under the terms of the Creative Commons Attribution 4.0 International license.

10.1128/mSystems.00677-20.7 TABLE S2 Fecal metabolites discriminating between model classes. Tables show significantly higher or lower fecal metabolites when comparing between groups. Model 1 (adherence to WHO healthy eating guidelines): controls only comparing individuals with a <35% adherence versus those with a ≥100% adherence to WHO healthy eating guidelines. Model 2 (exercise): controls with a ≥100% adherence versus athletes with a ≥100% adherence to WHO healthy eating guidelines. Model 3 (combined diet and exercise effect): controls with a <35% adherence versus athletes with a ≥100% adherence to WHO healthy eating guidelines. ^a^Multiplicity key is as follows: s, singlet; d, doublet; t, triplet; q, quartet; dd, doublet of doublets; m, multiplet. 1H shifts marked by an asterisk (*) were not confirmed experimentally due to the absence of unambiguous resonance values and are taken from the Human Metabolome Database (HMDB). Download Table S2, DOCX file, 0.04 MB. Copyright © 2020 Penney et al. 2020 Penney et al. This content is distributed under the terms of the Creative Commons Attribution 4.0 International license.

Targeted gas chromatography-mass spectrometry (GC-MS) of SCFA in feces revealed significantly higher levels of acetate (Benjamini-Hochberg false-discovery rate [pFDR] < 0.001), propionate (pFDR < 0.001), and butyrate (pFDR = 0.006) in healthy eating athletes relative to healthy eating controls (model 2; Fig. 2H, exercise effect). In addition to propionate (pFDR = 0.004) and butyrate (pFDR = 0.019), valerate (pFDR = 0.043) was found in higher concentrations in healthy eating athletes relative to controls with unhealthy eating profiles, while acetate was higher but not significant after Benjamini-Hochberg (BH) multiple testing corrections (pFDR = 0.056) (model 3; Fig. 2I, combined effect). There were no significant differences in the diet healthiness model (model 1; Fig. 2G) and no differences in isobutyrate or isovalerate concentrations in any model.

Targeted analysis of 10 organic acids in urine by GC-MS demonstrated lower levels of lactate (pFDR = 0.002) and 2-hydroxybutyrate (pFDR = 0.014) in healthy eating controls versus controls with unhealthy eating habits (model 1, healthy diet effect). 2-Methylbutyrate (pFDR = 0.043) was lower in healthy eating athletes versus healthy eating controls (model 2, exercise effect). In contrast to the fecal SCFA model, urinary acetate (pFDR = 0.006), propionate (pFDR = 0.003), 2-methylbutyrate (pFDR = 0.001), isovalerate (pFDR = 0.001), and lactate (pFDR < 0.001) were all lower in athletes with ≥100% adherence to healthy eating versus controls with <35% adherence to healthy eating (model 3, combined effect).

### Microbial diversity.

No difference in gut microbial diversity was found between comparative groups when investigating the effect of healthy eating (model 1; Fig. 2J) or exercise (Model 2; Fig. 2K). However, there was increased diversity of genera detected by 16S profiling in healthy eating athletes versus unhealthy eating controls, both in richness and evenness (model 3; Fig. 2L, combined effect) using Shannon (pFDR = 0.012), Simpson (pFDR = 0.020), whole-tree (phylogenic diversity [PD]; pFDR = 0.001), Chao1 (pFDR = 0.025), and observed feature (pFDR = 0.012) indices. Similar comparisons for each of the models with microbial metabolic pathways, species, and genera identified with metagenomic sequencing were not significantly different despite having similar trends (data not shown).

Linear regression analysis of ^1^H-NMR fecal water global profiles against Shannon, Simpson, and PD diversity indices, corrected for confounding factors (age, lean mass, and fat mass), revealed multiple metabolites associated with diversity (see Table S2). In contrast, no metabolites from urinary ^1^H-NMR global profiles were found to significantly correlate with measures of gut diversity. Fecal metabolites associated with higher diversity included increased short/medium-chain FAs valerate and caproate; branched SCFAs isobutyrate, isovalerate, and 2-methylbutyrate; branched-chain amino acid (BCAA) degradation products 2-oxoisocaproate and 2-oxoisovalerate; as well as products of phenylalanine (phenylacetate and 3-phenylpropionate), choline (dimethylamine), proline (2-methylproline), and uracil (ureidopropionate) metabolism. Increased diversity was also associated with lower glucose, isoleucine, asparagine, and histidine levels. See Table S2 for corrected *P* values (pFDR).

### Diet-gut microbiome-metabolome interactions.

**FIG 3:**
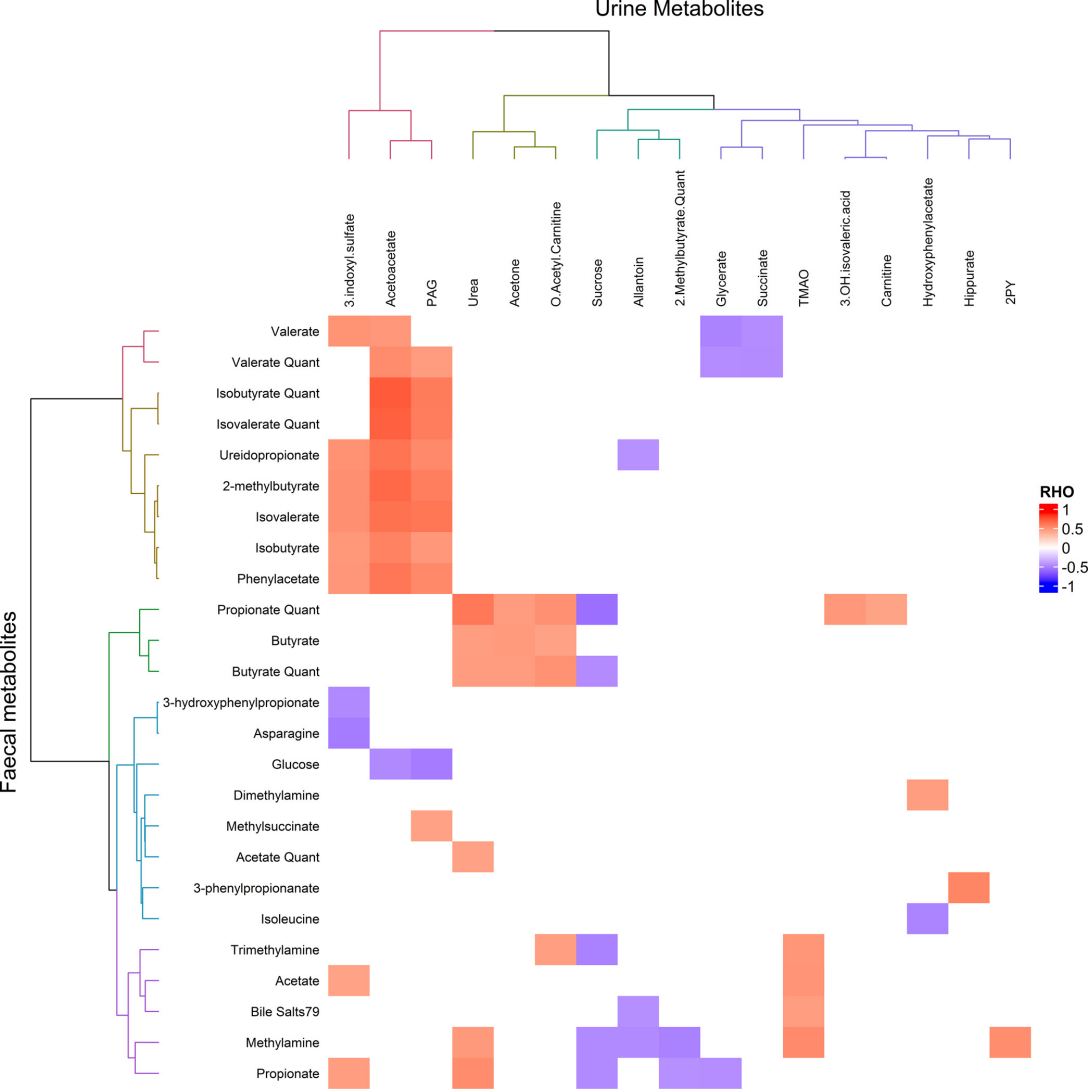
Fecal-urinary metabolic interactions. Significant Spearman correlations (pFDR, <0.05) between fecal and urinary metabolic data sets are shown, shaded according to the strength of the correlation coefficient (Rho). Correlations are clustered according to Euclidean distances.

**FIG 4:**
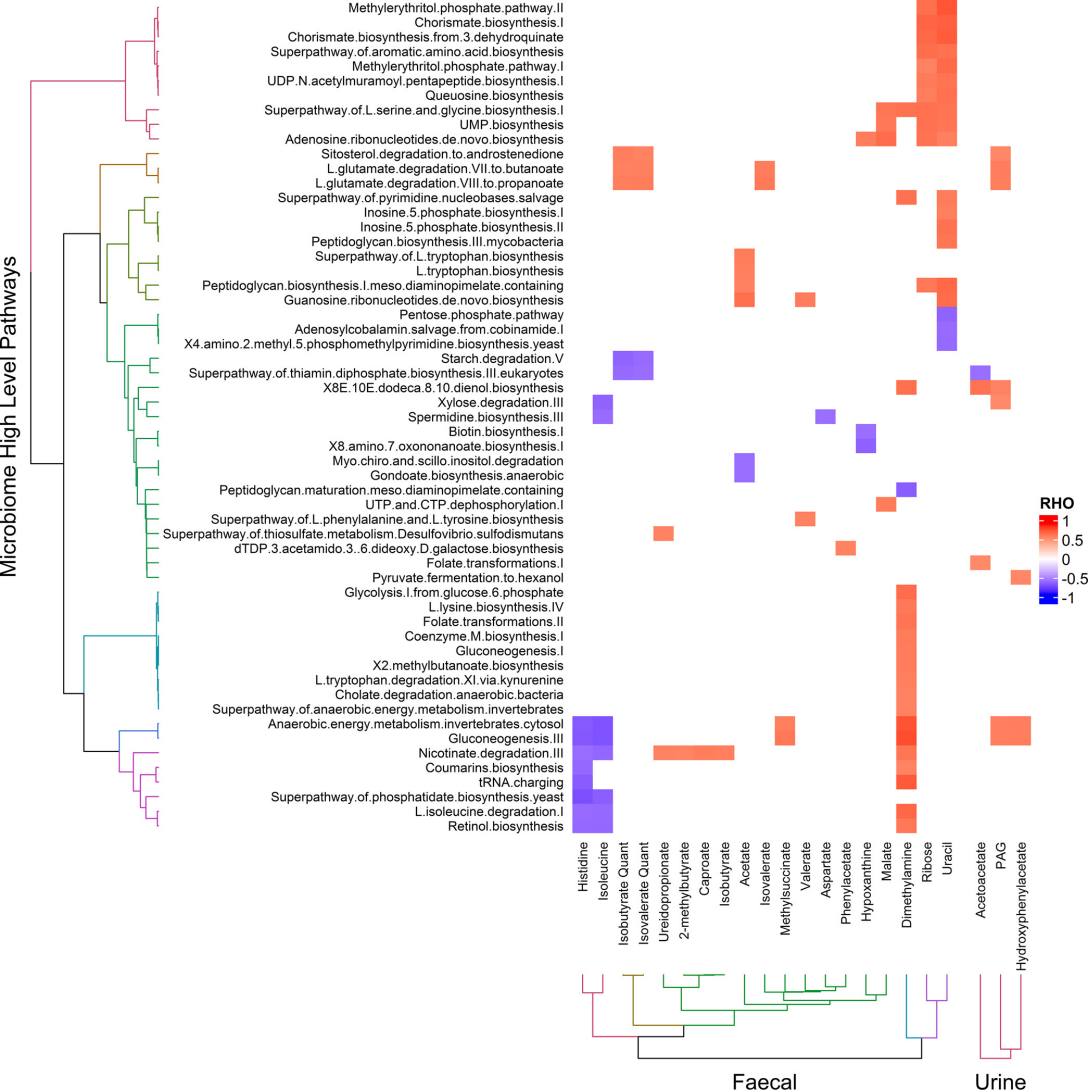
Microbial metabolic pathway-metabolite interactions. Significant Spearman correlations (pFDR, <0.01) between microbial metabolic pathways versus fecal and urinary metabolic data sets are shown, shaded according to the strength of the correlation coefficient (Rho). Correlations are clustered according to Euclidean distances.

A total of 47 fecal and 27 urinary metabolites of interest were subsequently quantified. Selection of these metabolites was based on discriminatory capacity (model weighting) from models 1 to 3 and/or metabolites associated with microbial diversity. A number of correlation matrices were generated using these quantified metabolites to explore gut metabolome-host metabolome interactions. First, fecal-urinary metabolome interactions were derived from Spearman correlations between the two data sets (see Fig. 3). Findings included the correlation of urinary 3IS with fecal SCFAs, urinary PAG with fecal phenylacetate, urinary hippurate with fecal 3-phenylpropionate, and urinary TMAO with fecal trimethylamine and methylamine. Second, Spearman correlations between metabolite levels and relative abundance of (i) microbial species (Fig. S1) and (ii) microbial metabolic pathways (Fig. 4) were explored, revealing numerous significant correlations (pFDR < 0.01 = 257 and 58 correlations, respectively). These included the correlation of urinary PAG levels with bacterial species from the *Actinobacteria* and *Proteobacteria* phyla as well as a number of bacterial metabolic pathways, including those related to gluconeogenesis, anaerobic energy metabolism, and glutamate degradation to SCFAs. We found a number of positive correlations between bacterial species and pathways with SCFAs, including the correlation of acetate with Roseburia hominis, Streptococcus suis, and Halobacteriovorax marinus, propionate with Leuconostoc citreum, and valerate with Pyrococcus horikoshii, Simkania negevensis, and Streptococcus suis. We identified a large number of species and pathways whose abundance correlated with fecal dimethylamine (DMA) excretion but which did not appear to influence TMAO levels. Whereas the heterofermentative lactic acid bacterium Leuconostoc citreum did correlate with higher TMAO levels. Interestingly, we also identified a number of lactococcal phages that correlated with TMAO. Furthermore, lactococcal phage levels correlated with increased TMAO:DMA and TMA:DMA ratios. Further correlations between dietary, metabolite, microbial pathway, and microbial diversity data sets were also performed (Fig. S2 to S5). Fecal butyrate correlated strongly with fiber intake, whereas propionate correlated most strongly with protein intake. Diversity was correlated with a number of metabolites, including valerate, medium-chain fatty acids (MCFA), branched SCFA, and branched-chain keto acids (BCKA), as well as lower amino acid levels. Increased diversity also correlated with a number of microbial pathways, including higher amino acid, BCAA, and nicotinate degradation, cofactor and amino acid biosynthesis, and gluconeogenesis pathways, along with a reduced activity in starch degradation, methionine, and fatty acid biosynthesis pathways.

10.1128/mSystems.00677-20.1 FIG S1 Gut microbial species-metabolite interactions. Significant Spearman’s correlations (pFDR, <0.01) between microbial species profiled by shotgun metagenomics sequencing versus fecal and urinary metabolic data sets are shown, shaded according to the strength of the correlation coefficient (Rho). Correlations are clustered according to Euclidean distances. Download FIG S1, TIF file, 10.4 MB. Copyright © 2020 Penney et al. 2020 Penney et al. This content is distributed under the terms of the Creative Commons Attribution 4.0 International license.

10.1128/mSystems.00677-20.2 FIG S2 Diet-metabolite interactions. Significant Spearman correlations (pFDR, <0.05) between diet versus fecal and urinary metabolic data sets are shown, shaded according to the strength of the correlation coefficient (Rho). Correlations are clustered according to Euclidean distances. Download FIG S2, TIF file, 3.8 MB. Copyright © 2020 Penney et al. 2020 Penney et al. This content is distributed under the terms of the Creative Commons Attribution 4.0 International license.

## DISCUSSION

Models of diet healthiness, exercise, or both each displayed metabolic and functional microbial signatures, with a number of discriminatory metabolites. Microbial diversity was associated with a combination of increased diet healthiness and exercise and also correlated with distinct microbially derived metabolites. Our results suggest that dietary changes alone did not significantly impact bacterial diversity, although they did affect gut microbial metabolites in urine and feces.

### Metabolic phenotype associated with diet and exercise.

As expected, the effect of adherence to WHO healthy eating guidelines was associated with higher markers of fruit and vegetable intake (urinary proline-betaine, formate, and hippurate in addition to lower sugar levels) (17, 22). Healthy eating was also characterized by lower urinary levels of lactate and 2-hydroxybutyrate, markers of anaerobic respiration and oxidative stress, respectively (22).

High levels of physical activity were linked with higher concentrations of *O*-acetyl carnitine, a metabolite associated with the intake of animal protein. Although protein consumption was higher in the athlete group and included whey protein supplementation in several individuals, higher *O*-acetyl carnitine may also have been due to increased fatty acid mobilization and oxidation (23). In addition, there were lower concentrations of urinary succinate in athletes, likely due to increased systemic utilization through the TCA cycle.

### Diet and exercise induced alterations in gut microbe-host cometabolism.

**Tryptophan metabolism.** In control participants assigned high healthy eating scores (model 1), we found lower levels of *N-*methylnicotinamide (NMND). NMND is generated from tryptophan metabolism through the kynurenine pathway in humans. The resulting nicotinamide is methylated by nicotinamide *N*-methyltransferase (NNMT), to form NMND, which is then further metabolized to 2-methyl-2-pyridone-5-carboxamide (2PY) (24). Nicotinamide serves an important role in energy regulation. Indeed, NNMT knockout in mice—resulting in higher nicotinamide and reduced NMND and 2PY—is protective of diet-induced obesity (25). Thus, the lower levels of NMND seen here suggest increased energy expenditure in healthy eaters.

Conversely, in the exercise effect model we find higher levels of urinary 2PY associated with physical activity (model 2). In addition, there is increased gut microbial metabolism of tryptophan through the indole pathway, leading to increased 3IS. This is a similar metabolic signature to that seen in malnourished children (24). Increased 2PY can be explained through indoleamine 2,3-dioxygenase (IDO)-mediated activation of the kynurenine pathway due to increased inflammation (8). Increased 3IS is likely due to a shift toward proteolytic fermentation of increased colonic protein by gut bacteria due to higher protein intake in healthy eating athletes relative to healthy eating controls (median intake, 240 g/day versus 104 g/day; *P* < 0.001). We also found that urinary 3IS was positively correlated with fecal SCFAs (Fig. 3), suggesting it was linked to increased fermentation of other substrates, including dietary fiber and complex carbohydrates.

**Phenylalanine metabolism.** Higher physical activity (model 2) and the combined effect of healthy diet and exercise (model 3) resulted in higher levels of urinary phenylacetylglutamine (PAG), produced through gut microbial conversion of phenylalanine to phenylacetate and subsequent conversion to PAG in the liver. In keeping, fecal phenylacetate was positively correlated with urinary PAG (Fig. 3), and we observed lower fecal phenylalanine and tyrosine concentrations in model 2. Consistent with these findings, PAG has previously been associated with a lean phenotype (26). Our study highlights a number of correlations between PAG levels and bacterial species and metabolic pathways. The ketone body acetoacetate was similarly associated with higher physical activity and the combined effect of healthy diet and exercise and can also be produced through ketogenic metabolism of phenylalanine.

While PAG was positively associated with higher physical activity (model 2), the more minor microbial phenylalanine metabolites 3-hydroxyphenylacetate (HPA) and 3-(3-hydroxyphenyl)-3-hydroxypropionic acid (HPHPA) were inversely correlated. Whereas healthy eating (model 1) was positively correlated with HPA levels in urine. The biological role of HPA and HPHPA is disputed. Some studies have associated HPA and HPHPA with neurological disorders (27). Others, however, have shown the contrary (28), and furthermore, HPA is thought to be a dietary marker of rutin intake, a flavonoid and antioxidant.

**Benzoate metabolism.** An alternative metabolic route for phenylalanine and other dietary aromatic compounds, such as catechin is via their metabolism to benzoate, which is further glycine conjugated predominantly in the liver to form hippurate (29). Here, we found higher urinary hippurate in individuals with a healthy diet score (model 1). Higher hippurate levels have been correlated with a lean phenotype and lower blood pressure as well as fruit intake (30, 31). In this study, urinary hippurate levels correlated strongly with fecal 3-phenylpropionate (Fig. 3), suggesting that the main source of hippurate production observed here was through microbial degradation of catechin from the diet.

**Choline metabolism.** We found higher levels of TMAO in both those that adhered to healthy eating guidelines (model 1) and those with high levels of physical activity (model 2) in addition to the combination of both. Circulating TMAO is thought to predict cardiovascular disease (CVD), possibly through altered cholesterol metabolism and oxidative stress (32, 33). However, the causative effect in humans is disputed and may be due to confounders such as kidney function and poor metabolic control (34). Furthermore, high concentrations of TMAO are present in the tissues of cold-water-dwelling fish, where it acts as an antifreeze agent, and is consequently also found in high levels in the urine of Japanese populations whose diet contains a high portion of fish and who do not have high risk for CVD (30). Moreover, TMAO was recently found to protect against impaired glucose tolerance and reduce endoplasmic reticulum stress (35).

As expected, urinary TMAO levels correlated with fecal trimethylamine and methylamine, although not with dimethylamine (DMA) (Fig. 3). Furthermore, TMAO correlated with markers of protein intake: urinary carnitine, acetyl-carnitine, and urea (Fig. S5), which is consistent with the higher reported intake of protein in the healthy eating athletes represented in the exercise and combined models. The microbial abundance data also demonstrated a dichotomy with respect to association between TMAO and DMA. We identified a large number of species and pathways whose abundance correlated with fecal DMA excretion but which did not appear to influence TMAO levels, whereas the heterofermentative lactic acid bacterium Leuconostoc citreum did correlate with higher TMAO levels. Intriguingly, the identification of a number of lactococcal phages that correlated with TMAO raises the possibility that changes to the gut environment, such as altered pH from changes to lactic acid bacteria, can alter levels of TMA/TMAO-producing and -metabolizing bacteria, leading to subsequent changes in host TMAO levels. Indeed, lactococcal phage levels also correlated with increased TMAO:DMA and TMA:DMA ratios, suggesting they may influence bacterial TMA dehydrogenase or TMAO aldolase activity.

**SCFA and branched-chain amino acid metabolism.** Fecal SCFAs were found in higher concentrations in participants with increased physical activity (model 2) and participants with combined healthy eating and increased exercise (model 3) relative to controls. SCFAs are produced through the bacterial fermentation of dietary fiber and complex carbohydrates. The most abundant SCFAs are acetate (C2), propionate (C3), and butyrate (C4). Valerate and the branched SCFAs isobutyrate, isovalerate, and 2-methyl-butyrate are produced by gut bacteria in smaller quantities (36). Butyrate is the preferred source of energy for colonic epithelial cells (37), whereas propionate, along with remaining butyrate, is used predominantly by hepatocytes for gluconeogenesis (38). Acetate is mainly utilized by muscle cells to generate energy. Importantly, SCFAs have been associated with reduced appetite and weight loss through stimulating release of the anorectic gut hormones peptide YY (PYY) and glucagon-like peptide-1 (GLP-1) (36, 39). Interestingly, it has been reported that undigested proteins and amino acids in the colon may serve as an additional substrate for SCFA production (40). Indeed, in this cohort, we found that while fecal butyrate correlated strongly with fiber intake, propionate correlated most strongly with protein intake, which is increased in healthy eating athletes (Fig. S2). We found a number of positive correlations between bacterial species and SCFAs (Fig. S1). These included the correlation of acetate with Roseburia hominis, Streptococcus suis, and Halobacteriovorax marinus, propionate with Leuconostoc citreum, and valerate with Pyrococcus horikoshii, Simkania negevensis, and Streptococcus suis.

Medium-chain fatty acids (MCFA), branched SCFA, and branched-chain keto acids (BCKA) were correlated with higher microbial diversity, seen after modeling the combined effects of healthy diet and exercise. The branched SCFAs isobutyrate, isovalerate, and 2-methylbutyrate are produced through the fermentation of BCAAs and have also been shown to modulate energy metabolism (41). Here, isobutyrate and isovalerate were both correlated with bacterial amino acid (glutamate) degradation pathways to propionate and butyrate and had an inverse correlation to pathways encoding starch degradation (Fig. 4). It is likely that bacteria were using similar pathways to ferment BCAAs in place of glutamate. Further, in fecal samples with higher diversity, we detected higher levels of 2-oxoisocaproate and 2-oxoisovalerate, BCKAs produced through the catabolism of the BCAAs leucine and valine, respectively. This initial transamination reaction, catalyzed by branched-chain aminotransferase, produces glutamate (also higher in fecal samples with higher diversity) and the respective BCKA (42). Subsequent leucine metabolism via 2-oxoisocaproate is ketogenic, while valine metabolism via 2-oxoisovalerate is glucogenic (43). This constellation of raised BCKAs, glutamate, and branched-SCFAs suggests that the increased diversity observed here was associated with an adaptation toward increased degradation of protein and amino acids in the colon. Additionally, we observed lower glucose and amino acid levels (histidine, asparagine, and isoleucine) with higher levels of diversity. It is not clear whether these changes were a result of microbial adaptation to a high protein/low sugar environment in the distal colon or if they were driven by an increase in microbial utilization of amino acids. Intriguingly, the lower fecal amino acid levels, seen here with higher microbial diversity, indicate that enhanced protein degradation increased beyond any increased protein intake. Corresponding changes were also noted in microbial metabolic pathways related to increased diversity, including higher abundance of higher amino acid, BCAA, and nicotinate degradation pathways (Fig. S3).

10.1128/mSystems.00677-20.3 FIG S3 Metagenomic pathway-diversity index correlations. Significant Spearman correlations (pFDR, <0.01) between microbial metabolic pathways and diversity indices (Shannon, Simpson, PD, Chao1) are shown, shaded according to the strength of the correlation coefficient (Rho). Correlations are clustered according to Euclidean distances. Download FIG S3, TIF file, 4.3 MB. Copyright © 2020 Penney et al. 2020 Penney et al. This content is distributed under the terms of the Creative Commons Attribution 4.0 International license.

In addition, SCFAs can also be utilized by bacteria for *de novo* amino acid biosynthesis (44). Here, increased fecal valerate correlated with increased exercise (models 2 and 3) and microbial diversity and was positively correlated with bacterial phenylalanine and tyrosine biosynthesis pathways (Fig. 4). Furthermore, increased fecal acetate also correlated with increased exercise (models 2 and 3) and was positively correlated with bacterial tryptophan biosynthesis pathways. Lastly, fecal acetate and valerate were positively associated with guanosine ribonucleotide biosynthesis pathways. These correlations suggest that SCFAs have an important role in enabling the biosynthesis of aromatic compounds.

### Conclusion.

We have shown that each exercise/diet group exhibited a distinct metabolic and functional microbial phenotype. The incorporation of an objective measure of dietary habits into the models enabled us to more accurately reduce the confounding effect of diet when investigating exercise and therefore better ascertain the individual biological sequelae of diet healthiness and exercise. Limiting the participants to males removed confounders resulting from gender. However, further studies are needed to confirm the reproducibility of these results in females. In addition, we were unable to isolate the effect of diet in participants with high physical activity levels due to a paucity of athletes that follow an unhealthy diet.

Although increased microbial diversity has previously been linked with health (45–48), increased diversity in the present cohort was associated with amplified proteolytic fermentation by gut bacteria. Since protein metabolism within the gut has been linked with the production of toxic compounds such as ammonia, amines, and sulfides (49, 50), increased gut bacterial diversity may include deleterious as well as beneficial effects on the host. This emphasizes the value of in-depth analysis at a metabolic pathway and taxonomic level in future microbiome studies.

## MATERIALS AND METHODS

### Study population.

Professional male athletes from the national Irish Rugby Football team (*n* = 40) and healthy controls (*n* = 46) matched for age and gender were enrolled in this study as previously described (8). Exclusion criteria included receiving antibiotics within 2 months before screening and prior diagnosis with any cardiovascular, gastrointestinal, or immunological condition. Control subgroups were established with body mass index (BMI) ranges matching the body types of the athletes. Approval was granted by the Cork Clinical Research Ethics Committee. Urine and fecal samples were collected from both groups and stored at −80°C until analysis.

### Acquisition of clinical exercise and dietary data.

Urine and fecal samples were collected from athletes while they attended an intensive training camp and from healthy but less active controls. Activity levels were validated using the EPIC-Norfolk questionnaire (51) and creatine kinase levels. Dietary intake was calculated from food frequency questionnaires (FFQ) administered by a research nutritionist (8).

### Gut microbiota analysis.

As determined previously, 16S taxonomic profiles had more robust characterization of taxa at and above the level of genus (7). Due to the removal of samples falling outside healthy diet thresholds, species and strain-level designations were unreliably varied within the study groups. As a result, the 16S data were used in order to more conservatively represent alpha diversity above the species level, whereas metagenomic sequencing was used to establish species and pathway data.

### DNA preparation.

Extraction and purification of DNA from fresh fecal samples were accomplished with the QIAmp DNA stool minikit (Qiagen, UK). The manufacturer’s protocol for the extraction kit was followed with the addition of a bead-beating step (30s × 3) to better disrupt cell walls. The resulting DNA was initially stored at −20°C prior to 16S rRNA sequencing before being stored at −80°C until the samples were prepared for shotgun metagenomics sequencing.

### 16S rRNA sequencing.

The complete procedures used for 16S rRNA gene amplicon sequencing were outlined previously (8). Briefly, 16S rRNA gene (v4) PCR amplicons were generated using a combination of universal 16S rRNA primers estimated to bind to 94.6% of all 16S genes. Sample-specific identifier tags and 454 adaptor sequences were combined with the primers. The AMPure magnetic bead purification system was used to clean both 16S amplicons and shotgun metagenomics libraries (Beckman Coulter; catalogue number 9A63880). The 16S amplicons were sequenced on a 454 genome sequencer FLX platform at the Teagasc sequencing facility using the manufacturer’s protocols. The 16S sequencing data were originally processed accordingly as follows: quality trimming of raw 16S sequences was done with the Qiime (v1.2) (52) software suite, using the SILVA 16S rRNA database (v106) (53). Reads were discarded from further analysis if they fell below a minimum quality score of 25 or were shorter than 150 bp. BLAST with default parameters was used with the SILVA database to generate input for MEtaGenome ANalyzer (MEGAN v4.70.4), which assigned reads to taxonomies. Following read clustering to operational taxonomical units (OTUs) and chimaera removal, Qiime was then used to generate measures of alpha diversity. Although the methods to process 16S sequencing data are continuously evolving, minimal changes have occurred with OTU assignment and the calculation of alpha diversity measures. Due to this, diversity indices as previously reported were used to describe alpha diversity above the species level (8).

### Shotgun metagenomic sequencing.

As described previously (7), metagenomic library preparation was performed with the Illumina Nextera XT DNA library preparation kit (Illumina, Inc., USA) in explicit accordance with the manufacturer’s protocol (15031942, Illumina). Prior to library preparation, DNA samples were normalized to 0.2 ng/μl using the Qubit v2.0 fluorometric quantification system (Thermo Fisher Scientific). Library fragment size was assessed with the Agilent 2100 bioanalyzer system (Agilent Technologies; catalogue number G2939BA). Finalized libraries were combined in equimolar concentration (2 nM) before sequencing. Metagenomic libraries were sequenced on the Illumina HiSeq 2500 (chemistry v4.0) next-generation sequencing (NGS) platform by Eurofins Genetic Services Ltd. (Ebersberg, Germany) using the high-output run mode for 2 × 125-bp paired-end reads with the addition of a PhiX library (1%) to estimate sequence quality. Contaminant reads from humans were removed from raw FASTQ sequence files with NCBI Best Match Tagger (BMTagger) software. Remaining reads were quality checked with Picard and SAMTools software, removing duplicate and substandard-quality reads. The resulting high-quality sequence data were subjected to functional profiling by the Human Microbiome Project Unified Metabolic Analysis Network (HUMAnN2 v.0.5.0) pipeline (54). Here, models of microbial metabolic pathways derived from the MetaCyc database were generated. Metagenomic taxonomic profiling was performed with the Kraken software package (v0.10.6) (55). Species-level shotgun metagenomics data were converted to relative abundance, and HUMAnN2 pathway profiles were normalized to copies per million units prior to statistical analysis.

### Metabolic profiling.

Metabolic analyses of urine and fecal biofluids were conducted using established ^1^H-NMR and GC-MS methods (56, 57), as previously described (7).

Global, untargeted ^1^H-NMR urine and fecal metabolic profiling analyses were performed on a 600-MHz spectrometer (Bruker BioSpin, Germany) using established methods (56, 58). Briefly, urine and fecal samples were prepared with a pH 7.4 phosphate buffer for ^1^H-NMR spectroscopy as described previously and analyzed at 300 K using the following standard one-dimensional pulse sequence with saturation of the water resonance: RD-g_z1_-90°-t_1_-90°-t_m_-g_z2_-90°-ACQ. The relaxation delay (RD) was set at 4 s, 90° represents the applied 90° radio frequency pulse, the interpulse delay (t_1_) was set to an interval of 4 μs, the mixing time (tm) was 10 ms, magnetic field gradients (g_z1_ and g_z2_) were applied for 1 ms, and the acquisition period (AQA) was 2.7 s. Water suppression was achieved through continuous wave irradiation at the water resonance frequency. Each spectrum was acquired using 4 dummy scans, 32 scans, and 64 K time domain points.

Targeted analysis to quantify SCFAs was conducted using GC-MS as previously described (57), using an Agilent 7890B gas chromatography system, equipped with an automatic liquid sampler, coupled to an Agilent 7000C single quadruple mass selective detector (Agilent Technologies, USA).

Urinary and fecal quality control samples were used for the nuclear magnetic resonance (NMR) and GC-MS analyses to ensure data quality.

### Objective assessment of dietary intake.

We applied a novel and validated mathematical tool capable of objectively assessing free-living individuals’ dietary patterns based on their urine composition, without the need to collect dietary data. The tool is based on a Monte Carlo cross-validated PLS-DA model built from global urinary metabolic profiles derived from a highly controlled environment (inpatient randomized controlled crossover trial) to ensure that healthy participants were fully adherent to four dietary interventions that reflected four different levels of adherence (25, 50, 75, and 100%) to WHO healthy eating recommendations (increased fruits, vegetables, whole grains, and dietary fiber and decreased fats, sugars, and salt) (17). The application of this model has been successfully validated in free-living populations (17, 59). ^1^H-NMR urinary metabolic profiles from athletes and controls were projected into the MCCV-PLS-DA model to calculate a predicted score for each participant that reflected their adherence to healthy eating. We considered a score that is predicted at 100% or higher to be reflective of healthy eating; e.g., the global profile of such a score has (on average) higher concentrations of biomarkers reflective of the 100% diet than trial participants following the 100% diet.

### Relationship between self-reported dietary intake and independent urinary metabolite assessment.

Based on the self-reported food frequency questionnaires (FFQ), there were no significant differences in the frequency of citrus fruit or fruit and vegetable consumption comparing controls with a ≤35% adherence to healthy eating versus controls with a ≥100% adherence to healthy eating (citrus consumption, *P = *0.96; fruits and vegetables, *P = *0.67) or controls with a ≤35% adherence to healthy eating versus athletes with a ≥100% adherence to healthy eating (citrus consumption, *P = *0.85; fruits and vegetables, *P = *0.77).

However, we and others have demonstrated the use of urinary biomarkers to independently assess the consumption of specific food groups, including proline-betaine for citrus fruits (20, 60) and hippurate for fruits and vegetables (31, 61). Investigation of urinary proline-betaine and hippurate found higher intake of citrus and fruits and vegetables in the healthy eating athletes and controls. This suggests a level of misreporting in the self-reported data that makes it difficult to interpret. Therefore, participants’ dietary intakes were scored using the validated metabolic profiling dietary assessment tool that relies on urinary metabolic profiles and dietary biomarkers instead of self-reported FFQ (17).

### Statistical analysis.

^1^H-NMR spectra were phased and digitized over the range δ0 · 5 to 9 · 5 and imported into MATLAB to undergo median fold change normalization (62). Multivariate statistical analysis was performed using SIMCA v14.1 (Umetrics) (62). The three comparative groups previously defined were modeled using OPLS-DA: (i) controls with a <35% adherence to healthy eating versus controls with a ≥100% adherence to healthy eating (model 1), (ii) controls with a ≥100% adherence to healthy eating versus athletes with a ≥100% adherence to healthy eating (model 2), and (iii) controls with a <35% adherence to healthy eating versus athletes with a ≥100% adherence to healthy eating (model 3). The OPLS-DA models were established based on one predictive component and one orthogonal component. Unit variance scaling was applied to ^1^H-NMR spectral data. The fit and predictability of the models obtained were determined by the R^2^_Y_ and Q^2^_Y_ values, respectively. Significant metabolites differentiating between groups were obtained from ^1^H-NMR OPLS-DA models after investigating ^1^H-NMR signals with correlation coefficient values higher than 0.4. Jack-knifed 95% confidence intervals of the coefficients were used to confirm the significance of the variables.

Univariate statistical analysis (two-sided Mann-Whitney U test) was used to identify discriminatory metabolites in SCFA quantitative data sets. *P* values were adjusted for multiple testing using the Benjamini-Hochberg (BH) false-discovery rate method (pFDR).

Linear regression analysis of ^1^H-NMR global spectra was performed against Shannon, Simpson, and whole-tree bacterial diversity indices, corrected for confounding factors (age, lean mass, and fat mass), using an in-house MATLAB (release 2014a) script. Metabolites with adjusted pFDR values of <0.01, were considered significant and were subsequently visualized in a Manhattan plot.

Finally, heat maps showing significant Spearman correlations between data sets were generated using the ComplexHeatmap script in R (63). Spearman correlations were first calculated in MATLAB. Correlations between metabolite-metabolite data sets with a pFDR <0.05 were included. Due to the large number of variables, a false-discovery rate of 1% was set for correlations, including microbiome taxa and pathway data sets. Hierarchical clustering of correlations was performed using Euclidean distances.

### Metabolite identification.

A combination of data-driven strategies such as such as SubseT Optimization by Reference Matching (STORM) (64) and Statistical TOtal Correlation SpectroscopY (STOCSY) (65) and analytical identification strategies were used to aid structural identification of significant discriminatory metabolites. Specifically, a catalogue of 1D ^1^H-NMR sequences with water presaturation and 2D NMR experiments such as J-Resolved spectroscopy, ^1^H-^1^H TOtal Correlation SpectroscopY (TOCSY), ^1^H-^1^H COrrelation SpectroscopY (COSY), ^1^H-^13^C Hetero-nuclear Single Quantum Coherence (HSQC) and ^1^H-^13^C Hetero-nuclear Multiple-Bond Correlation (HMBC) spectroscopy were performed. Finally, when possible, metabolites were confirmed by *in situ* spiking experiments using authentic chemical standards.

### Metabolite quantification.

Semiquantification of identified metabolites from ^1^H-NMR profiles was performed using an in-house MATLAB script to annotate maximal intensity of median fold change normalized spectral peaks. The highest-intensity spectral peak was identified for each measured metabolite and precisely quantified for each individual. Manual annotation enabled precise marking of peaks, allowing for variations in chemical shift in individual samples. Where spectral peaks overlapped with other metabolites, the highest-intensity unambiguous spectral region was used.

### Data availability.

All presented data are tabulated and detailed in the main text and supporting information. Codes used to analyze these data are referenced in the main text. Metagenomic sequencing data are available at the European Nucleotide Archive repository (accession number PRJEB15388). The 16S rRNA sequence reads are available from the Sequence Read Archive (accession number PRJEB4609).

10.1128/mSystems.00677-20.4 FIG S4 Correlation between fecal metabolites. Significant Spearman correlations (pFDR, <0.05) within the fecal metabolite data set are shown, shaded according to the strength of the correlation coefficient (Rho). Correlations are clustered according to Euclidean distances. Download FIG S4, TIF file, 4.9 MB. Copyright © 2020 Penney et al. 2020 Penney et al. This content is distributed under the terms of the Creative Commons Attribution 4.0 International license.

10.1128/mSystems.00677-20.5 FIG S5 Correlation between urine metabolites. Significant Spearman correlations (pFDR, <0.05) within the urine metabolite data set are shown, shaded according to the strength of the correlation coefficient (Rho). Correlations are clustered according to Euclidean distances. Download FIG S5, TIF file, 3.5 MB. Copyright © 2020 Penney et al. 2020 Penney et al. This content is distributed under the terms of the Creative Commons Attribution 4.0 International license.
