# Supplementary material for: Investigating the Role of Diet and Exercise in Gut Microbe-Host Cometabolism
Source: mSystems. 2020 Dec 1;5(6):e00677-20. doi: 10.1128/mSystems.00677-20 (PMC7716389; doi:10.1128/mSystems.00677-20)
Supplement: TABLE S1 [file mSystems.00677-20-st001.docx]

### Supplementary Table 1: Urinary metabolites discriminating between model classes

|  | | | |
| --- | --- | --- | --- |
| **<35% controls Vs >100% control [Model 1 – Diet healthiness]** | | | |
| **Metabolite** | **^1^H chemical shift δ (multiplicity)^a^** | **Association** | **Source / Role** |
| Acetone | 2.24 (s) | **↑** | Ketone |
| Citrate *[UV]* | 2.55~ (d), 2.69~ (d) | **↑** | Fruit / TCA cycle |
| Creatinine | 3.05 (s), 4.06 (s) | **↑** | Muscle mass |
| TMAO | 3.27 (s) | **↑** | Choline metabolism, fish, meat |
| Proline Betaine | 3.30 (s), 3.11 (s) | **↑** | Citrus fruits |
| 3-Hydroxyphenylacetate | 3.48(s), 6.79 (m), 6.81 (m), 6.86 (m), 7.26 (t) | **↑** | Gut microbial - Tyrosine metabolism |
| Hippurate | 7.55 (t), 7.64 (t), 7.84 (d), 3.98 (d) | **↑** | Fruits & vegetables |
| Formate | 8.46 (s) | **↑** | Fruits & vegetables |
| Lactate | Targeted GC-MS | **↓** | Anaerobic metabolism |
| 2-hydroxybutyrate | Targeted GC-MS | **↓** | Glutathione synthesis |
| N-methylnicotinamide (NMND) | 4.486 (s), 8.19 (t), 8.90 (d), 8.97 (d), 9.28 (s) | **↓** | Vit B3 / Endogenous Tryptophan metabolism |
| Pyroglutamate | 2.04 (m), 2.41 (m), 2.51 (m), 4.18 (q) | **↓** | Glutamate metabolism |
| Sucrose | 3.49 (t), 3.58 (dd), 3.79 (t), 3.84 (m), 3.91 (m), 4.06 (t), 4.23 (d), 5.42 (d) | **↓** | Sugars |
| **>100% controls Vs >100% athletes [Model 2 - Exercise]** | | | |
| Acetoacetate | 2.29 (s), 3.45 (s) | **↑** | Ketone |
| Phenylacetylglutamine (PAG) | 2.11 (m), 2.27 (m), 3.67 (m), 4.19 (m), 7.36 (t), 7.43 (t) | **↑** | Gut microbial – Phenylalanine metabolism |
| Carnitine | 3.23 (s), 2.45 (dd), 3.43 (m) | **↑** | Red meat |
| N-methylnicotinamide | 4.486 (s), 8.19 (t), 8.90 (d), 8.97 (d), 9.28 (s) | **↑** | Niacin (B3) |
| 3-Indoxyl sulfate *[UV]* | 7.51(d), 7.71 (d), 7.21 (m), 7.28 (m) | **↑** | Gut microbial – Tryptophan metabolism |
| TMAO | 3.27 (s) | **↑** | Choline metabolism, fish, meat |
| 2 methyl 2 pyridone 5 carboxamide (2PY) | 3.65 (d), 6.67 (d), 7.83 (dd), 8.34 (d) | **↑** | NAD degradation, Tryptophan metabolism |
| O-Acetyl Carnitine | 3.19 (s), 2.15 (s) | **↑** | Red meat |
| 2-methylbutyrate | Targeted GC-MS | **↓** | Branched SCFA |
| Succinate | 2.41 (s) | **↓** | TCA cycle |
| Citrate *[UV]* | 2.55~ (d), 2.69~ (d) | **↓** | Fruit / TCA cycle |
| 3-Hydroxyphenylacetate | 3.48(s), 6.79 (m), 6.81 (m), 6.86 (m), 7.26 (t) | **↓** | Gut microbial / Tyrosine metabolism |
| Allantoin | 5.39 (s) | **↓** | Gut microbial – oxidative stress |
| 3-(3-hydroxyphenyl)-3-hydroxypropionic acid (HPHPA) | 2.62 (dd), 2.71 (dd)*, 5.02 (dd), 6.85 (dd), 6.91 (t), 6.98 (m), 7.30 (t) | **↓** | Gut microbial - dysbiosis |
| **<35% controls Vs >100% athletes [Model 3 – Diet healthiness & Exercise]** | | | |
| Acetoacetate | 2.29 (s), 3.45 (s) | **↑** | Ketone |
| Phenylacetylglutamine (PAG) | 2.11 (m), 2.27 (m), 3.67 (m), 4.19 (m), 7.36 (t), 7.43 (t) | **↑** | Gut microbial – Phenylalanine metabolism |
| Carnitine | 3.23 (s), 2.45 (dd), 3.43 (m) | **↑** | Red meat |
| TMAO | 3.27 (s) | **↑** | Choline metabolism, fish, meat |
| Proline Betaine | 3.30 (s), 3.11 (s) | **↑** | Citrus fruits |
| 3-Indoxyl sulfate | 7.51(d), 7.71 (d), 7.21 (m), 7.28 (m) | **↑** | Gut microbial |
| Hippurate | 7.55 (t), 7.64 (t), 7.84 (d), 3.98 (d) | **↑** | Fruits & vegetables |
| 2 methyl 2 pyridone 5 carboxamide (2PY) | 3.65 (d), 6.67 (d), 7.83 (dd), 8.34 (d) | **↑** | NAD degradation, Tryptophan metabolism |
| *O*-Acetyl Carnitine | 3.19 (s), 2.15 (s) | **↑** | Red meat |
| Acetate | Targeted GC-MS | **↓** | SCFA |
| Propionate | Targeted GC-MS | **↓** | SCFA |
| 2-methylbutyrate | Targeted GC-MS | **↓** | Branched SCFA |
| Isovalerate | Targeted GC-MS | **↓** | Branched SCFA |
| Lactate | Targeted GC-MS | **↓** | Anaerobic metabolism |
| Alanine | 1.49 (d), 3.79 (q) | **↓** | Amino acid |
| Pyroglutamate | 2.04 (m), 2.41 (m), 2.51 (m), 4.18 (q) | **↓** | Glutamate metabolism |
| Sucrose | 3.49 (t), 3.58 (dd), 3.79 (t), 3.84 (m), 3.91 (m), 4.06 (t), 4.23 (d), 5.42 (d) | **↓** | Sugars |
| Allantoin | 5.39 (s) | **↓** | Gut microbial – oxidative stress |
