# Supplementary material for: Investigating the Role of Diet and Exercise in Gut Microbe-Host Cometabolism
Source: mSystems. 2020 Dec 1;5(6):e00677-20. doi: 10.1128/mSystems.00677-20 (PMC7716389; doi:10.1128/mSystems.00677-20)
Supplement: TABLE S2 [file mSystems.00677-20-st002.docx]

### Supplementary Table 2: Faecal metabolites discriminating between model classes

|  | | | |
| --- | --- | --- | --- |
| **<35% controls Vs >100% control [Model 1 – Diet healthiness]** | | | |
| **Metabolite** | **^1^H chemical shift δ (multiplicity)^a^** | **Association** | **Source / Role** |
| SMCSO metabolite 1^(^[^1^](#_ENREF_1)^,^ [^2^](#_ENREF_2)^)^ | 2.78 (s) | **↑** | Cruciferous vegetables |
| SMCSO metabolite 2^(^[^1^](#_ENREF_1)^,^ [^2^](#_ENREF_2)^)^ | 2.81 (s) | **↑** | Cruciferous vegetables |
| S-methyl-L-cysteine-sulfoxide (SMCSO) | 2.83 (s) | **↑** | Cruciferous vegetables |
| 3-hydroxyphenylpropionate | 2.47 (t), 2.84 (t), 6.75 (m), 6.81 (t), 6.87 (m), 7.25 (t) | **↑** | Gut microbial / Caffeine |
| Bile Salts 73 | 0.73 (s) | **↓** | Bile acids |
| 2-aminobutyrate | 0.98 (t) | **↓** | Glutathione synthesis |
| Methylsuccinate (citramalate) | 1.09 (d), 2.13 (dd)*, 2.53 (dd), 2.63 (m)* | **↓** | Microbial, malate analogue |
| 2-hydroxybutyrate | 1.20 (d) | **↓** | Glutathione synthesis |
| Acetate | 1.92 (s) | **↓** | SCFA |
| *N*-acetylglucosamine | 2.06 (s), 3.47-3.85 (m), 5.21 (d) | **↓** | Amino sugar, bacterial cell wall constituent |
| Beta-alanine | 2.56 (t), 3.18 (t) | **↓** | Beta amino acid  Uracil metabolism |
| Ribose | 3.52 (dd), 4.11 (t), 4.93 (d), 5.25 (d) +… | **↓** | Monosaccharide, RNA constituent |
| Hypoxanthine | 8.21 (d) | **↓** | Purine derivative |
| **>100% controls Vs >100% athletes [Model 2 - Exercise]** | | | |
| Bile salts 79 | 0.79 (s) | **↑** | Bile acids |
| Valerate | 0.89 (t), 1.30 (m), 1.54 (m), 2.18 (t) | **↑** | SCFA |
| Butyrate | 0.90 (t), 1.56 (m), 2.16 (t) + Targeted GC-MS | **↑** | SCFA |
| Propionate | 1.06 (t), 2.19 (q) + Targeted GC-MS | **↑** | SCFA |
| Threonine | 1.33 (d), 3.59 (d), 4.26 (dqt) | **↑** | Amino acid |
| Acetate | 1.92 (s) + Targeted GC-MS | **↑** | SCFA |
| Methylamine | 2.60 (s) | **↑** | Choline metabolism |
| Trimethylamine | 2.88 (s) | **↑** | Choline metabolism |
| Uracil | 5.81 (d), 7.55 (d) | **↑** | RNA nucleobase |
| Anserine | 7.17 (s), 8.35 (s), 3.65 (s) | **↑** | Dipeptide |
| 3-hydroxyphenylpropionate | 2.47 (t), 2.84 (t), 6.75 (m), 6.81 (t), 6.87 (m), 7.25 (t) | **↓** | Gut microbial / Caffeine |
| Dimethylamine | 2.73 (s) | **↓** | Choline metabolism |
| Glycine | 3.56 (s) | **↓** | Amino acid |
| Phenylalanine | 3.20 (dd), 3.29 (dd), 3.99 (dd), 7.34 (d), 7.38 (m), 7.43 (m) | **↓** | Amino acid |
| Tyrosine | 6.91 (d), 7.21 (d) | **↓** | Amino acid |
| **<35% controls Vs >100% athletes [Model 3 – Diet healthiness & Exercise]** | | | |
| Acetate | 1.92 (s) | **↑** | SCFA |
| Anserine | 7.17 (s), 8.35 (s), 3.65 (s) | **↑** | Dipeptide |
| Bile salts 79 | 0.79 (s) | **↑** | Bile acids |
| Butyrate | 0.90 (t), 1.56 (m), 2.16 (t) + Targeted GC-MS | **↑** | SCFA |
| Methylamine | 2.60 (s) | **↑** | Choline metabolism |
| Propionate | 1.06 (t), 2.19 (q) + Targeted GC-MS | **↑** | SCFA |
| Trimethylamine | 2.88 (s) | **↑** | Choline metabolism |
| Valerate | 0.89 (t), 1.30 (m), 1.54 (m), 2.18 (t) + Targeted GC-MS | **↑** | SCFA |
| 2-hydroxybutyrate | 1.20 (d) | **↓** | Glutathione synthesis |
| Proline | 2.01 (m), 2.35 (m), 4.14 (dd) | **↓** | Amino acid |
| *N*-acetylglucosamine | 2.06 (s), 3.47-3.85 (m), 5.21 (d) | **↓** | Amino sugar, bacterial cell wall constituent |
| Glycine | 3.56 (s) | **↓** | Amino acid |
| **Bacterial diversity (Shannon / Simpson / Whole Tree) regression [Model 4 - Diversity]** | | | |
| Bile Salts 76 | 0.76 (s) | **↑ pFDR = 0.015** | Bile acids |
| 2-methylbutyrate | 0.86 (t), 1.05 (d), 1.39 (m), 1.5 (m), 2.21 (m) | **↑ pFDR = 0.011** | Branched SCFA |
| Caproate | 0.86 (t), 1.27 (m), 1.53 (m), 2.17 (t) | **↑ pFDR = 0.016** | MCFA |
| Valerate | 0.89 (t), 1.30 (m), 1.54 (m), 2.18 (t) | **↑ pFDR = 0.012** | SCFA |
| Isovalerate | 0.91 (d), 1.96 (m), 2.06 (d) | **↑ pFDR = 0.012** | Branched SCFA |
| α-ketoisocaproate (2-oxoisocaproate) | 0.94 (d), 2.10 (m), 2.62 (d) | **↑ pFDR = 0.028** | BCAA degradation |
| Isobutyrate | 1.07 (d), 2.35 (m) | **↑ pFDR = 0.011** | Branched SCFA |
| α-ketoisovalerate (2-oxoisovalerate) | 1.13 (d), 3.02 (m) | **↑ pFDR = 0.028** | BCAA degradation |
| Glutamate | 2.05 (m), 2.12 (m), 2.35 (m), 3.75 (dd) | **↑ pFDR = 0.019** | Amino acid |
| Malate | 2.36 (dd), 2.67 (dd), 4.32 (dd) | **↑ pFDR = 0.021** | TCA cycle |
| Ureidopropionate | 2.39 (t), 3.29 (q)* | **↑ pFDR = 0.003** | Uracil metabolism |
| 3-phenylpropionate (Hydrocinnamate) | 2.49 (t), 2.90 (t), 7.30 (m) | **↑ pFDR = 0.016** | Phenylalanine metabolism |
| Dimethylamine | 2.73 (s) | **↑ pFDR = 0.001** | Choline metabolism |
| 2-methylproline | 2.75 (s), [3.60-61 (s)] | **↑ pFDR = 0.002** | Arginine & Proline metabolism |
| Phenylacetate | 3.54 (s), 7.31 (m), 7.39 (m) | **↑ pFDR = 0.028** | Phenylalanine metabolism |
| Isoleucine | 1.02 (d), 3.68 (d) | **↓ pFDR = 0.010** | BCAA |
| Asparagine | 2.95 (dd), 2.87 (dd), 4.01 (dd) | **↓ pFDR = 0.005** | Amino acid |
| Glucose | 3.25 (dd), 3.42 (m), 3.49 (m), 3.54 (dd), 3.74 (m), 3.84 (m), 3.91 (dd), 4.65 (d), 5.24 (d) | **↓ pFDR = 0.009** | Sugars |
| Uridine | 3.80 (dd)*, 3.91 (dd)*, 4.12 (m)*, 4.22 (dd)*, 4.34 (dd)*, 5.91 (d), 5.92 (d), 7.88 (d) | **↓ pFDR = 0.009** | Nucleoside |
| Histidine | 7.11 (s), 7.93 (s) | **↓ pFDR = 0.001** | Amino acid |
| Nicotinate | 7.53 (dd)*, 8.26 (m), 8.62 (dd), 8.94 (d) | **↓ pFDR = 0.030** | Vit B3 |

## References

1. Garcia-Perez I, Posma JM, Gibson R, Chambers ES, Hansen TH, Vestergaard H, Hansen T, Beckmann M, Pedersen O, Elliott P, Stamler J, Nicholson JK, Draper J, Mathers JC, Holmes E, Frost G. 2017. Objective assessment of dietary patterns by use of metabolic phenotyping: a randomised, controlled, crossover trial. Lancet Diabetes Endocrinol 5:184-195.

2. Edmands WM, Beckonert OP, Stella C, Campbell A, Lake BG, Lindon JC, Holmes E, Gooderham NJ. 2011. Identification of human urinary biomarkers of cruciferous vegetable consumption by metabonomic profiling. J Proteome Res 10:4513-21.
